# Fluid Therapy in Dogs and Cats With Sepsis

**DOI:** 10.3389/fvets.2021.622127

**Published:** 2021-02-25

**Authors:** Federico Montealegre, Bridget M. Lyons

**Affiliations:** ^1^Department of Medical and Scientific Affairs, Nova Biomedical, Waltham, MA, United States; ^2^Department of Veterinary Medicine and Surgery, University of Missouri, Columbia, MO, United States

**Keywords:** sepsis, septic shock (MeSH), fluid therapy, critical care—small animal, immunopathogenesis

## Abstract

Sepsis is currently defined as life-threatening organ dysfunction caused by a dysregulated host response to infection. Sepsis may occur secondary to infection anywhere in the body, and its pathogenesis is complex and not yet fully understood. Variations in the host immune response result in diverse clinical manifestations, which complicates clinical recognition and fluid therapy both in humans and veterinary species. Septic shock is a subset of sepsis in which particularly profound circulatory, cellular, and metabolic abnormalities are associated with a greater risk of mortality than with sepsis alone. Although septic shock is a form of distributive shock, septic patients frequently present with hypovolemic and cardiogenic shock as well, further complicating fluid therapy decisions. The goals of this review are to discuss the clinical recognition of sepsis in dogs and cats, the basic mechanisms of its pathogenesis as it affects hemodynamic function, and considerations for fluid therapy. Important pathophysiologic changes, such as cellular interaction, microvascular alterations, damage to the endothelial glycocalyx, hypoalbuminemia, and immune paralysis will be also reviewed. The advantages and disadvantages of treatment with crystalloids, natural and synthetic colloids, and blood products will be discussed. Current recommendations for evaluating fluid responsiveness and the timing of vasopressor therapy will also be considered. Where available, the veterinary literature will be used to guide recommendations.

## Introduction

Sepsis is one of the most important causes of morbidity and mortality in human medicine and is associated with high mortality rates in dogs and cats. Globally, more than 30 million human patients are diagnosed with sepsis each year; 5 million of them die, with a high economic burden and long-term morbidity affecting the survivors (1, 2). The prevalence and incidence of sepsis in veterinary medicine are not well-documented. Available data show that in dogs, mortality rates associated with septic peritonitis range from 21 to 68% (3–5). Other reports indicate mortality rates of 70% for dogs that experience organ failure (6). In cats with sepsis, mortality rates of 40% have been documented (7). Based on these results and others, it appears likely that sepsis is a major cause of mortality in hospitalized dogs and cats.

Vascular alterations, damage to the endothelial glycocalyx, hypoalbuminemia, and myocardial dysfunction are important pathophysiologic consequences of sepsis and complicate decision-making in regards to fluid therapy. Patients may present with varying degrees of illness severity, and may or may not manifest all of these pathophysiologic consequences of sepsis. Ultimately, fluid therapy in these patients must tailored to each individual, taking into account their relative intravascular volume, interstitial hydration, fluid responsiveness, and cardiac function.

## Definition

Sepsis is defined as life-threatening organ dysfunction caused by a dysregulated host response to infection (1). It may occur with infection anywhere in the body, and may be secondary to bacterial, fungal, or viral agents. Septic shock is further defined as a subset of sepsis in which particularly profound circulatory, cellular, and metabolic abnormalities are associated with a greater risk of mortality than with sepsis alone (1). These definitions represent our most current understanding of sepsis and accompany a change in the clinical recognition of sepsis in human medicine. Prior to 2016, sepsis was clinically recognized by the presence or suspicion of infection together with at least 2 out of 4 systemic inflammatory response syndrome (SIRS) criteria (Table 1). The SIRS criteria may be fulfilled secondary to a multitude of causes, with infection being only one of them. In human patients with sepsis, the SIRS criteria have been found to lack both sensitivity and specificity for organ dysfunction, and a cutoff value of 2 does not necessarily represent an increased risk of mortality (8). Concerns over the failure of the SIRS criteria to adequately identify patients with infections that have increased risk of mortality, as well as the focus of these criteria on inflammation as opposed to a dysregulated host response, have prompted new clinical criteria for sepsis. Sepsis is now recognized as the presence or suspicion of infection in conjunction with evidence of organ dysfunction per the sequential organ failure assessment (SOFA) score (Table 2), and the “quick” SOFA (qSOFA) score (Table 3) has been proposed as a screening tool for sepsis in patients housed outside of the ICU (1). Septic shock is clinically recognized by the need for vasopressors to maintain normotension or by a serum lactate >2 mmol/L in patients without hypovolemia (1). Significant controversy regarding these changes exists in human medicine, particularly surrounding the use of qSOFA, and whether or not updated definitions are needed in veterinary medicine is an open question. As of this time, the SIRS criteria are still used to diagnose sepsis in dogs and cats, as no consensus regarding a change in the clinical recognition of sepsis has been reached in veterinary medicine. The SIRS criteria in dogs and cats were respectively derived from a cohort of surgical patients and a small necropsy study, and may not be representative of all small animal patients with sepsis (9, 10). That said, the presence of SIRS in dogs and cats should raise the practitioner's index of suspicion that sepsis may be present; however, if a patient is clinically deteriorating yet does not meet the requisite number of SIRS criteria, sepsis should still be considered as a differential.

**Table 1:** The systemic inflammatory response syndrome (SIRS) criteria in dogs, cats, and humans.

	**Dogs (2/4 required)**	**Cats (3/4 required)**	**Humans (2/4 required)**
Temperature (°F)	<100.6 or >102.6	<100.0 or >104.0	<96.8 or >100.4
Heart rate (beats/min)	>120	<140 or >225	>90
Respiratory rate (breaths/min)	>20	>40	>20
White blood cell (×10^3^/μl); % bands	<6 or >16; >3%	<5 or >19	<4 or >12; 10%

**Table 2:** The sequential organ failure assessment (SOFA) score in humans.

	**0**	**1**	**2**	**3**	**4**
PaO_2_/FiO_2_ ratio	≥400	<400	<300	<200 with respiratory support	<100 with respiratory support
Platelets (×10^3^/μl)	≥150	<150	<100	<50	<20
Bilirubin (mg/dL)	<1.2	1.2–1.9	2.0–5.9	6.0–11.9	>12.0
Cardiovascular	MAP ≥70 mmHg	MAP <70 mmHg	Dopamine <5 or dobutamine (any dose)	Dopamine 5.1–15 or epinephrine ≤0.1 or norepinephrine ≤0.1	Dopamine >15 or epinephrine >0.1 or norepinephrine >0.1
Glasgow Coma Scale	15	13–14	10–12	6–9	<6
Creatinine (mg/dl) Urine output (ml/day)	<1.2	1.2–1.9	2.0–3.4	3.5–4.9 <500	>5.0 <200

*Each parameters is given a score of 0–4, so that the total score can range from 0 to 24*.

**Table 3:** The “quick” SOFA (qSOFA) score in humans.

**Criteria**	**Points**
Respiratory rate ≥22/min	1
Change in mental status	1
Systolic blood pressure ≤100 mmHg	1

*If the cutoff for each parameters is met, the patient is assigned 1 point, so that the total score can range from 0-3 points. A score of ≥2 indicates organ dysfunction*.

## Pathophysiology of Sepsis

The following sections on pathophysiology will briefly focus on the most important abnormalities leading to organ failure and fluid leakage during sepsis and septic shock, including the receptors involved in the activation of the inflammatory response and the complement cascade, neutrophil and platelet dysfunction, and immunosuppression. The microcirculatory and hemodynamic abnormalities involving endothelial cells will be addressed in detail elsewhere (Chapter 4).

### Initiation of the Inflammatory Response

Fundamentally, the host immune response is designed to localize, confine, and destroy pathogens while repair mechanisms are activated. Sepsis is a dysregulated form of this response, and alterations can manifest as endothelial cell damage, coagulopathies, microcirculatory and hemodynamic alterations, metabolic and neuroendocrine immune network abnormalities, endoplasmic reticulum stress, autophagy, and many other pathophysiological processes leading to vascular leakage and organ failure. Early in the inflammatory response, the innate immune system is activated upon the recognition of pathogen and host cell molecules by a subset of germline-encoded pattern recognition receptors (PRRs) (11, 12). These receptors can be found in the extracellular and subcellular compartments, such as the cellular and endosomal membranes and the cytosol; in secreted forms present in the bloodstream and interstitial fluids; and in intracellular vesicles, such endosomes and lysosomes (13, 14). The sensing of microorganisms by PRRs, regardless of their location, relies on recognizing molecules frequently found in pathogens or host cell products. The PRR superfamily has four major sub-families: the toll-like receptors (TLRs); NOD-like receptors; the retinoic acid-inducible gene 1 (RIG-1)–like receptors; and the C-type lectin receptors (15). Pattern recognition receptors engage pathogen-associated molecular patterns (PAMPs), which are conserved structures in invading organisms that serve to identify these organisms as foreign, or damage-associated molecular patterns (DAMPs), which are endogenous molecules, such as mitochondria that are released or modified by sterile insults, such as trauma (14, 16–18). Circulating nucleic acids, either DNA or RNA derived from pathogens or host cells, are also strong activators of PRRs (19). Intracellular PRRs are activated by signaling cascades originated by extracellular PRRs (20). During a response, nucleic acids are an important target for innate immune recognition, as all microbial pathogens depend on their DNA and/or RNA for replication. Cells and other sensor systems of the innate immune system expressing PRRs include neutrophils (21), macrophages (22), dendritic cells (23), platelets (24), complement system (25), natural killer cells (26), fibroblasts (27), and some epithelial cells, such as those of the intestinal tract (28). In a host, cells of the immune system expressing PRRs, as well as those of sensor systems, such as the complement system, can initiate both proinflammatory and anti-inflammatory responses. The intracellular signaling process is complex, with numerous signaling pathways, ultimately leading to multi-gene expression, which is involved in adaptive immunity and inflammation. Recent data on gene activation during sepsis show that in human septic patients, a total of 910 unique genes are differentially expressed (29). This study also revealed that 43 functional cellular pathways that were closely associated with inflammation and response to infection were activated during sepsis. The inflammatory response is an essential step in activating the immune system to the presence of infection so that cells from the innate response can rapidly locate and mount an effective defense against pathogens (30). This defense is typically well-organized, with the inflammation under control after the infection is resolved. After this, the immune system can prime itself for an effective immune response to future infection (31). If the response becomes dysregulated, both the hyperinflammatory and the hypoinflammatory responses can lead to many of the sequela that are observed in sepsis and septic shock. Among these are disseminated intravascular coagulation (DIC), multi-organ dysfunction, and peripheral vasodilation leading to hypotension and subsequent organ hypoperfusion (32, 33).

### Local and Systemic Inflammatory Response

The host–pathogen interactions at the site of injury or infection, neutrophils, resident macrophages, and dendritic cells coordinate the initial inflammatory response through PAMP–PRR and DAMP–PRR signaling. Downstream, this pathway activates the inflammasome response, a multiprotein intracellular complex that includes enzymes, such as caspase-1 (34). The inflammasome pathway in neutrophils and macrophages triggers the production and secretion of the highly proinflammatory cytokines interleukin-1β (IL-1β) and IL-18 (35). During activation, neutrophils become autophagic, and other cell-death mechanisms are also activated and integrated into the host's defense against bacteria (36). These mechanisms regulate the generation of the proinflammatory immune response (37). Pathogen- and damage-associated molecular patterns may also directly activate receptors on neutrophils, which induce migration and recruitment into infection sites (38). Neutrophils—key cells in sepsis—display a wide range of effector mechanisms to counteract pathogens, which include phagocytosis, the production of reactive oxygen species, and killing of ingested pathogens by proteases (39). Activated neutrophils release neutrophil extracellular traps (NETs), which are webs of DNA and antimicrobial proteins designed to kill pathogens and aid in pathogen clearance (40). Excessive NET formation promotes inflammation and tissue damage during sepsis. Exosomes also play an important role in amplifying the inflammatory response. Exosomes are extracellular vesicles released by cells, such as neutrophils, macrophages, and platelets, and participate in local and systemic intercellular communication (41). The proinflammatory effects of exosomes are mediated by cytokines (e.g., IL-1α, IL-1β, IL-6, IL-18, TNF-α), miRNA, DAMPs, and NETs contained within the vesicles. The vesicles activate neutrophils, macrophages, and endothelial cells. Macrophages interact with bacterial products, as previously described for neutrophils, and release proinflammatory cytokines (e.g., TNF-α and IL-6), which contribute to vascular endothelial dysfunction, increased vessel diameter, and leakiness, the latter leading to fluid and immune cell infiltration of the surrounding tissues (42). The excessive amounts of proinflammatory cytokines produced by these cells lead to a hyperinflammatory state called the cytokine storm (43). This process is part of the dysregulation of the immune system during sepsis and is characterized by high levels of circulating IL-6 and IL-10 and the decreased ability of monocytes, macrophages, and dendritic cells to produce hyperinflammatory cytokines, such as TNF-α and interferon gamma (IFN-γ) in response to PAMPs and DAMPs. Current evidence suggests that in dogs with sepsis, there is a dysregulated proinflammatory cytokine production. In one study, numerous cytokine levels were measured in dogs with sepsis and compared to healthy dogs and those with non-infectious systemic inflammation (44). The results showed that the levels of the pro-inflammatory cytokines IL-6 and IL-8 and several chemokines (CXCL-8, keratinocyte-derived chemokine, and CCL2), and high-mobility group box-1 (HMGB-1) were higher in dogs with sepsis than in the healthy controls. An interesting finding in the study was that CCL2 was found to be predictive of poor survival. It should be pointed out that HMGB-1 is a DAMP that activates the innate responses of TLR2 and TLR4 (45). In humans with sepsis, the presence of HMGB-1 has been associated with poor outcomes (46). Another study compared levels of proinflammatory cytokines in dogs with naturally-occurring *Babesia rossi* infection to healthy controls and found that dogs with babesiosis experienced a similar proinflammatory cytokine storm, which correlated with the disease severity (47).

### Sepsis and Septic Shock

There are two basic components in sepsis, which combine to form a continuum of events between hyper-inflammation and hypo-inflammation. The role of neutrophils and platelets during the development of sepsis and septic shock as well as the most important mechanisms leading to immunosuppression will be addressed. Sepsis develops when the immune response to an infection becomes generalized and involves normal tissues remote from the site of an injury or infection. It is characterized by a systemic dysregulated host response to infection in which there are simultaneous hyperinflammatory and hypo-inflammatory states driven by the dysfunction of both the innate and adaptive immunity. An early hyperinflammatory phase in which the cytokine storm progresses is accompanied by the compensatory anti-inflammatory response syndrome to limit the tissue damage (48). How the early and acute hyperinflammatory phase transitions to the chronic or late hypo-inflammatory phase as sepsis progresses to septic shock is not fully understood. What is known is that some of the most important determinants of immunoparalysis are macrophage deactivation, negative regulatory mediators, such as programmed cell death-1 (PD-1), increased apoptosis (with a decrease in T cell counts), increased suppression of immune cells, increased production of anti-inflammatory cytokines, and alterations in energy production leading to cell anergy (49). During the late phase of sepsis and in septic shock, there are numerous hemodynamic, microcirculatory, cellular, and metabolic abnormalities that cause increased mortality and morbidity in affected patients (50). In general terms, the most important circulatory damage leading to multi-organ dysfunction is induced in part by the activation of the complement cascade and impairment in both platelets and neutrophils, including the excessive production of NETs (51). Dysregulated activation of the complement cascade, which is a tightly regulated multiprotein complex and a major component of the innate immune response, is designed to detect, destroy, and clear invading pathogens as well as amplifying the inflammatory response (52). Pathogen destruction is mediated by the lytic complement component—the membrane attack complex (MAC), which also causes injury to endothelial cells (53). Under normal conditions, bystander cells have a protective mechanism against the MAC: CD59 (54). With downregulation of CD59 and dysfunctional regulators, there is excessive activation of the MAC, causing bystander endothelial cells in the microcirculatory space to be damaged and inducing multi-organ dysfunction (55–57).

During septic shock, neutrophils play a pivotal role. They experience highly dysregulated functions, including the recruitment of immature cells, impaired migration, inefficient pathogen recognition (leading to impaired bacterial control), tissue damage, and cell margination (58). These alterations trigger an endothelial cell dysfunction that leads to increased permeability and fluid leakage from the intravascular space to the interstitium (59). Neutrophils lose elasticity and become more rigid, resulting in the altering of their migration through endothelial tight junctions (60). This excess rigidity in the cells is responsible for capillary occlusion, which itself is involved in multiple organ failure (59). Organ damage is enhanced as the lifespan of neutrophils is increased compared to those of neutrophils associated with non-septic inflammation, and these mature neutrophils are less likely to return to the bone marrow, promoting the margination of dysfunctional cells in the capillary networks (61). Neutrophils produce excessive NETs, which, in turn, activate the coagulation cascade, leading to immunothrombosis (62).

Immunothrombosis is the interaction between coagulation and innate immunity that triggers a generalized disseminated intravascular coagulation (DIC). It is important to note that there are other factors involved in coagulation in sepsis, such as monocytes and macrophages activated by invading microorganisms and their components (63). Coagulation activation also becomes dysfunctional (64). Additionally, during septic shock, inflammation stimulates capillary sequestration *of neutrophils* by activating circulating forms of these cells, which cells are responsible for further occlusion of the small vessels as well as the migration of vital organs and the release of cytotoxic substances (proinflammatory cytokines, proteolytic enzymes) that are involved in tissue damage and multiple organ failure, as shown in a study conducted by Mantovani et al. (65).

Platelets also are important contributors to organ failure during septic shock, as they actively participate in microvascular and mitochondrial dysfunction, DIC, acute kidney injury, and cardiac dysfunction (66). Recently published data show that activated platelets have a dual role in the immune response. First, they can engage the innate mechanisms of defense by directly killing pathogens as well as facilitating pathogen clearance by activating macrophages and neutrophils. Second, they promote NETs with the formation of platelet aggregates and disseminate microthrombi, which can be a risk factor for mortality (67). As a consequence, platelets promote endothelial damage, thrombosis, and organ failure. There are several mechanisms by which platelets can induce microvascular dysfunction during septic shock. In addition, they possess procoagulant properties that result in widespread microvascular coagulation and sepsis-induced disseminated intravascular coagulation. The nature of the thrombo-inflammatory response lies in the intense cross talk that takes place between platelets, neutrophils, and NETs and that results in widespread intravascular coagulation, microvascular occlusion, and ischemic and cytotoxic organ damage (68).

A state of induced immunosuppression normally predominates over the proinflammatory response, often starting during the early onset of septic shock (69). There are several mechanisms involved in the induction of the immunosuppression. These include a dysregulated compensatory anti-inflammatory response (70), increased apoptosis of T and B cells (lymphocyte exhaustion) (71), immune paralysis (increased tolerance to endotoxins) (72), and dysregulated expansion of T-regulatory cells. *Immune paralysis* refers to a state consisting of the reduced ability to trigger an immune response to a pathogen. Macrophages have decreased expression of antigen-presenting capabilities, with a reduction of important monokines that are required for an immune response (72). Another late feature of septic shock is the reprogramming of macrophages to an anti-inflammatory M2 phenotype, all of which is an attempt to decrease inflammation but which results in defective pathogen destruction caused by phagocytic cells (73). During septic shock, lymphocyte exhaustion occurs as the effector function of T and B cells is impaired. There is sustained and increased expression of inhibitory immune checkpoint molecules on exhausted T-cell surfaces and an increased expression of the PD-1 receptor and ligand (PD-L1) (63). Recent studies have demonstrated that peripheral blood polymorphonuclear neutrophils (PMNs) from septic patients express high levels of PD-L1 (74). These cells exhibit a decline in their capacity to phagocytize bacteria; therefore, sepsis drives altered PMN function. A consequence, the syndrome contributes to the increased risk of secondary and opportunistic infections and late death associated with sepsis and septic shock (70).

## Implications for Fluid Therapy

### Generation of Cardiovascular Alterations

Cardiovascular dysfunction in septic shock involves both peripheral vascular dysfunction and myocardial alterations. There are several mechanisms involved, from dilation of the veins and arteries to the dysregulated innate immune response affecting the endothelium and microcirculation. One mechanism in particular is the increased permeability of the intravascular space to the interstitium leading to tissue fluid loss and relative hypovolemia, which reduces ventricular diastolic function (75). It has been documented in humans that sepsis-induced myocardial dysfunction occurs in about 25–50% of patients with septic shock (76). The activation of the Toll-like receptors by PAMPs/DAMPs triggers the inflammatory cascade, as described in the preceding sections (77, 78). Once the inflammasome has been activated, the production of inflammatory cytokines is triggered. The release of chemotactic cytokines and intercellular adhesion molecules, such as ICAM-1 attracts neutrophils and macrophages from the microvessels to the myocardium, where they adhere (79, 80). Cell activation and the inflammatory response impact the coordination of the depolarization of the cardiomyocytes and subsequent calcium release, leading to decreased cardiomyocyte contractility. Complement activation also plays a key role in septic cardiomyopathy. Complement fragment 5a (C5a) is a potent anaphylatoxin (81). The presence of dysregulated amounts of C5a activates receptors C5aR1 and C5aR2 (present in cardiac myocytes), resulting in defective action potentials. In addition, alterations are induced by C5a, such as changes in the intracellular calcium homeostasis and electrophysiological functions that, when induced, result in defective action potentials in cardiomyocytes and the activation of neutrophils leading to dysregulated NET production (82). It should be pointed out that the NETs are composed of histones, which also cause defective action potentials, and the buildup of reactive oxygen species. The activated complement cascade will generate a membrane complex attack, which causes pore formation in cells, both in pathogens as well as in hosts cells, such as endothelial cells. This action will trigger endothelial dysfunction, including the production of microthrombi and DIC (57).

### Vascular Alterations

Sepsis results in a loss of vasomotor tone in the resistance vessels, resulting hypotension despite normal or increased cardiac output. This sepsis-induced vasoplegia affects both the arterial and venous circulation. Arterial vasodilation results in a relative hypovolemia and subsequent hypotension, reducing oxygen delivery to tissues. Venodilation occurs in the splanchnic and cutaneous vascular beds, resulting in an increase in unstressed volume and consequent decreased venous return and cardiac output (83). In addition, endothelial damage and microvascular thrombosis results in a reduced functional capillary density and abnormal microcirculatory flow (57). The ultimate goal of fluid therapy in patients with poor perfusion is to increase tissue oxygen delivery; however, these vascular alterations result in a maldistribution of blood flow. Although fluid resuscitation may increase the stressed volume in these patients and result in improvement in macrocirculatory parameters (e.g., blood pressure), microcirculatory alterations may persist and the goal of increasing oxygen delivery may remain unfulfilled.

### Endothelial Glycocalyx

The endothelial glycocalyx is a gel-like substance comprised of proteoglycans, glycoproteins, and glycosaminoglycans that lines the endothelium and regulates vascular permeability. The endothelial glycocalyx also plays a role in vascular relaxation by transmitting shear stress to endothelial cells which then produce nitric oxide, thus contributing to vasodilation (84). See Chapter 4 for further discussion of the functions of the endothelial glycocalyx.

In patients with sepsis, the endothelial glycocalyx is thought to be degraded, and this degradation has been associated with increases in TNF-α, IL-1β, IL-6, and IL-10 (84). Thinning of the endothelial glycocalyx results in alterations in vascular permeability, resulting in intravascular fluid loss and tissue edema. Additionally, fluid therapy may further damage the endothelial glycocalyx and hypervolemia has been associated with degradation in experimental models (84). However, a human study of patients with sepsis found that total crystalloid volume infused was not associated with shedding of an endothelial glycocalyx component (syndecan-1), suggesting that fluid volume does not contribute to endothelial glycocalyx damage in sepsis (85).

Albumin is not contained within the endothelial glycocalyx as it is repelled by the negatively-charged glycosaminoglycans, but does accumulate on the luminal surface (86). When the endothelial glycocalyx is damaged, this layer of albumin is reduced as well, further contributing to a decrease in capillary oncotic pressure. Evidence suggests that administration of albumin-containing fluids (e.g., serum albumin solutions or fresh frozen plasma) may be protective to the endothelial glycocalyx as albumin carries erythrocyte-derived sphingosine-1-phosphate to the endothelium, where it suppressed matrix metalloproteinase activity (84).

### Hypoalbuminemia

Hypoalbuminemia occurs in sepsis for a plethora of reasons, including downregulated hepatic production, gastrointestinal and/or renal losses, decreased protein intake, and leak through a damaged endothelial barrier. Our improved understanding of microvascular function and the revised Starling's forces (see Chapter 2) have led to the appreciation that there is no resorption of fluid at the venule end of the capillary, the so-called “no absorption rule.” Thus, the old paradigm of administering colloids (natural or synthetic) to hypoalbuminemic patients to reduce edema and “pull” fluid into the vasculature no longer holds true. Although the administration of colloid solutions may not redistribute fluid into the intravascular space, hypoalbuminemia decreases the capillary oncotic pressure and may also contribute to endothelial glycocalyx damage, both of which may worsen interstitial edema.

### Myocardial Dysfunction

Sepsis-induced myocardial dysfunction has been well-documented in humans, and is characterized by decreased systolic function (87). Typically when there is decreased contractility, there is diastolic compensation, and the left ventricular end diastolic volume is increased, which helps to preserve stroke volume and therefore cardiac output. However, in many human patients with sepsis, there is decreased diastolic compliance as well (87). It is unclear why some patients with sepsis develop diastolic dysfunction, but it is associated with increased morbidity (87). Patients that have myocardial dysfunction may be less responsive to fluid therapy, and patients with decreased contractility may require administration of positive inotropes to increase cardiac output. These patients may also be less tolerant of intravenous fluids and prone to congestive heart failure, requiring more caution with fluid therapy.

## Current Recommendations

### Fluid Resuscitation

Patients with sepsis frequently present with hypotension, which may be secondary to relative hypovolemia from vasodilation, absolute hypovolemia from gastrointestinal or renal losses, or myocardial dysfunction. Fluid therapy is considered a cornerstone of sepsis treatment, in addition to antimicrobials, source control, and frequently, vasopressors. The complexity of the disease process combined with variation in illness severity and presentation have made the formulation of guidelines that apply to all patients difficult, and clinically it is often challenging to determine how much fluid should administered.

One of the most well-known trials that addressed resuscitation in human patients with sepsis is the Early Goal Directed Therapy (EGDT) trial by Rivers et al. (88). This single-center study randomized 263 patients with sepsis or septic shock to receive either EGDT or standard care for the first 6 h of hospitalization (87). Patients in both groups received a combination of IV fluids and vasopressors to achieve a mean arterial pressure (MAP) of 65 mmHg or greater, a central venous pressure (CVP) of 8–12 mmHg, and a urine output (UOP) of 0.5 ml/kg/h or greater (87). Patients in the EGDT group also had resuscitation endpoints of a central venous oxygen saturation (ScvO_2_) ≥70% and a hematocrit ≥30%, and a dobutamine infusion was used to increase cardiac output if CVP, MAP, and hematocrit were optimized by ScvO_2_ remained lower than 70% (87). Patients in the EGDT group received more IV fluids, blood transfusions, and dobutamine, and had a significantly lower mortality rate compared to the standard care group (30.5 vs. 46.5%, *p* = 0.009) (87). These findings led to widespread adoption of protocolized EGDT, and in 2004 EGDT was added to the Surviving Sepsis Campaign (SSC) guidelines (89). However, since the publication of this study, three larger multicenter trials comparing a protocolized EGDT to standard care have been performed: ProCESS, ARISE, and ProMISE (90–92). These trials failed to show a mortality benefit of EGDT over standard care; however, in each of these trials illness severity was lower and patients were enrolled later in hospitalization compared to the Rivers trial, which may in part explain why the protocolized care was less effective. In veterinary medicine, a resuscitation protocol using blood pressure, ScvO_2_, CVP, lactate, base deficit, and hematocrit was evaluated in 30 dogs with sepsis or septic shock secondary to pyometra (93). ScvO_2_ and base deficit were the best predictors of non-survival, but this protocol has not been compared to other resuscitation strategies to determine if there is a mortality benefit of using such a protocol (93).

The current SSC guidelines recommend administration of 30 ml/kg of crystalloid within the first hour of resuscitation of sepsis-induced hypoperfusion, as evidenced by organ dysfunction, hypotension, or increased lactate (94, 95). This volume of fluid has a low quality of evidence to support it, and was adopted primarily because it is considered usual practice in the early stages of resuscitation (93). Indeed, these recommendations have been met with some controversy (96). It is difficult to recommend this as a standard in veterinary medicine, given the lack of evidence for this volume of fluid in humans as well as species-specific differences in blood volume and fluid tolerance. Given the heterogeneity of clinical presentations and varying degrees of concurrent hypovolemia in cats and dogs, an individualized, patient-based approach would appear reasonable. In patients with evidence of hypoperfusion and without concurrent cardiac disease, a strategy of incremental isotonic crystalloid fluid boluses (10–15 ml/kg) can be used, with reassessment of the patient's status to determine if they are fluid responsive. Dynamic measures of fluid responsiveness, such as the passive leg raise (PLR) have proven to be better predictors of fluid responsiveness than static parameters, such as CVP and ScvO_2_; however, PLR has not been adopted in veterinary medicine due to the anatomic differences between humans and dogs and cats (97). Other dynamic variables used to identify fluid responsiveness, such as pulse pressure variation and systolic pressure variation are useful in patients undergoing mechanical ventilation with a set tidal volume, but have little use in the majority of veterinary patients. For further discussion of monitoring for fluid responsiveness, see Chapter 19.

If the patient is not responsive to IV fluids and remains hypotensive, administration of a vasoactive medication is indicated. Norepinephrine (0.1–1 mcg/kg/min) is the vasopressor of choice in the treatment of septic shock, and early use in resuscitation has been associated with improved hemodynamics (83, 89, 98). Although definitions of early administration vary, recent evidence in humans with septic shock suggests that administration of norepinephrine within the first few hours of septic shock confers a mortality benefit (99). Norepinephrine acts on both α- and β-adrenergic receptors, although its actions on α-adrenergic receptors predominate, causing primarily vasoconstriction. This not only increases arterial vascular tone, but causes venoconstriction as well, resulting in a decrease in unstressed volume and an increase in venous return and cardiac output (99). Evidence suggests that norepinephrine may also improve microvascular flow in septic shock (100). If decreased systolic function secondary to myocardial depression is present, dobutamine (5–15 mcg/kg/min) can also be considered as a positive inotrope.

### Maintenance

Following resuscitation, many patients with sepsis require ongoing intravenous fluid therapy due to both decreased intake as well as ongoing losses, particularly if the gastrointestinal tract is the source of sepsis. The goal of ongoing fluid therapy is to maintain euhydration, which may be difficult. This is in part due to altered vascular permeability and hypoalbuminemia, both of which contribute to increased leak of fluid from the intravascular to the interstitial space. Additionally, critically ill patients frequently experience increased ADH secretion, resulting in fluid retention (101). In human patients with sepsis, net positive fluid balance is common and each 1L of cumulative fluids at 72 h of hospitalization has been associated with an increased odds of mortality (102). Individual patient fluid needs should be assessed frequently (every 4–6 h), using physical examination and body weight. Clinical signs of fluid overload include increasing body weight, serous nasal discharge, increased respiratory rate and effort, peripheral edema, body cavity effusions, chemosis, and jugular venous distention. If point-of-care ultrasound is available, serial monitoring for the presence of effusions, B-lines indicating alveolar-interstitial syndrome, and left atrial enlargement can be extremely useful in identifying fluid overload prior to the onset of clinical signs. For further discussion of fluid overload, see Chapter 20.

Although enteral water is never appropriate for treatment of hypoperfusion, it may be used to provide maintenance water to patients that no longer require resuscitation. If the patient is willing to drink, water can be provided by mouth. In patients that are unwilling or unable to drink and are unable to tolerate IV fluids, a nasogastric tube can be placed to administer water. Any water that is provided via the enteral route should be measured and recorded, so that it can be factored into a comprehensive fluid plan.

### Fluid Types

#### Crystalloids

Crystalloid solutions are the mainstay of fluid therapy in patients with sepsis, and balanced solutions are preferred over unbalanced solutions. One of the first large trials comparing balanced crystalloids (lactated Ringer's solution [LRS] and Plasmalyte A) to 0.9% sodium chloride in 15,802 critically ill humans was the SMART trial (103). A secondary analysis of 1,641 patients with sepsis in this trial found that patients in the balanced crystalloid group had lower mortality (26.3 vs. 31.2%, adjusted odds ratio 0.74; 95% confidence interval [CI], 0.59–0.93; *p* = 0.01), a greater number of vasopressor-free days (20 ± 12 vs. 19 ± 13; aOR, 1.25; 95% CI: 1.02–1.54), and a lower incidence of adverse renal events within 30 days (35.4 vs. 40.1%; aOR, 0.78; 95% CI: 0.63–0.97) (104). Although this effect is seen in patients without sepsis, it appears to be greater in patients with sepsis (102). Administration of high chloride fluids, such as 0.9% sodium chloride is thought to induce renal vasoconstriction and subsequent acute kidney injury (105). High chloride fluids have also been shown in experimental sepsis models to increase TNF, IL-10, and IL-6 concentrations and to impair microcirculatory function compared to balanced crystalloids (106, 107). The underlying pathogenesis of these differences is an ongoing area of investigation.

#### Synthetic Colloids

The use of synthetic colloids, such as hydroxyethyl starch (HES) is controversial, and has generally fallen out of favor in patients with sepsis. The VISEP trial evaluated 537 human patients with sepsis and found that there was a higher rate of acute kidney injury and need for renal replacement therapy in patients treated with 10% HES (200/0.5) compared to LRS (108). Conversely, the CRYSTMAS trial compared 6% HES (130/0.4) to 0.9% sodium chloride in 196 human patients with sepsis and found no difference in renal values or mortality between the groups; however, this study was small and may have been underpowered to detect a difference (109). The 6S trial compared 6% HES (130/0.42) to Ringer's acetate in 798 patients with sepsis and found increased 90 day mortality as well as increased need for renal replacement therapy in the HES group (110). A Cochrane review of HES compared to other fluid therapies concluded that there is an increased risk of acute kidney injury and need for renal replacement therapy in all patients treated with sepsis, and an increased risk of renal injury based on RIFLE (Risk, Injury, Failure, Loss of renal function, End-stage kidney disease) criteria in septic vs. non-septic patients (111). It is difficult to know if this information can be extrapolated to veterinary patients, as there are species-specific differences in HES metabolism. In addition, dogs and cats with sepsis are typically hospitalized for shorter durations compared to human patients, and receive far less total colloid. Several retrospective studies have evaluated HES use in dogs with conflicting results, although none have specifically evaluated a population of dogs with sepsis (112–114). Two retrospective studies in non-azotemic cats failed to show an association between HES administration and acute kidney injury or mortality (115, 116). In one of these studies, cats with sepsis were specifically evaluated and there was no HES-associated acute kidney injury noted; only 14 cats were in this group and the authors cautioned against making recommendations until a larger group of septic cats could be evaluated (115). Given that there is no clear survival advantage for synthetic colloids in patients with sepsis and there is potential risk of renal injury, use of these fluids in this patient population is not recommended. For further discussion of the controversies surrounding synthetic colloids, see Chapter 7.

#### Albumin

Hypoalbuminemia is common in patients with sepsis, and has been associated with poor prognosis in both humans and dogs (3, 117, 118). Despite this, it is unclear whether this is a marker of disease severity or a contributor to morbidity and mortality. It is also unclear whether or not albumin transfusion results in improved outcomes, although evidence suggests that it may improve mortality in patients with septic shock. Several human studies have evaluated the effect of albumin transfusion in critically ill patients. The SAFE study was a multicenter, randomized, double blind trial comparing 4% human serum albumin (HSA) to 0.9% sodium chloride over 28 days in 6,997 human ICU patients (119). Overall, there was no difference in mortality, organ failure, or length of stay between the groups (116). However, in the subset of patients with sepsis, there was a trend toward decreased mortality in patients that received albumin compared to 0.9% sodium chloride (30.7 vs. 35.5%, RR 0.87, 95% CI: 0.74–1.02, *p* = 0.09) (116). As a result of this finding, the ALBIOS trial was conducted. This trial was a multicenter, randomized, open label comparison of 20% albumin and crystalloid compared to crystalloid alone in 1,818 adults with sepsis or septic shock (120). Patients that received albumin had a higher mean arterial pressure, a shorter time on vasopressors, and lower net fluid balance. Overall there was no difference in mortality or organ failure between the groups, but a *post-hoc* analysis of the 1,121 patients with septic shock did find lower mortality in the albumin group (RR 0.87, 95% CI: 0.77–0.99, *p* = 0.03), indicating that there may be a benefit to albumin transfusion in this patient population (117).

Evidence supporting albumin transfusion in veterinary medicine is less robust. A retrospective comparison of 25% HSA transfusion to no albumin in 39 dogs with septic peritonitis found that survivors had increasing albumin levels during hospitalization, but albumin transfusion itself was not associated with survival (116). A prospective, randomized trial of 5% canine albumin compared to clinician-directed therapy in 14 dogs with septic peritonitis found that albumin levels remained increased 24 h after transfusion in the albumin group, but the study was not powered to evaluate mortality as an outcome (121). Given the evidence available in humans, it is reasonable to administer albumin to patients in septic shock if crystalloids alone are insufficient for maintaining intravascular volume. Due to the potential for acute and delayed transfusion reactions when administering xenotransfusions, administration of canine albumin is preferable to HSA in dogs. As no species-specific albumin product exists for cats and there is no evidence supporting or opposing albumin transfusion in this species, no recommendations can be given.

#### Blood Products

In the Rivers trial, patients were transfused with packed red blood cells (pRBCs) to maintain a minimum hematocrit of 30% (87). Due to the adoption of EGDT as standard of care, this transfusion threshold became widespread. In 2014, the TRISS trial was published, which compared a conservative transfusion threshold (hemoglobin <7 g/dl) to a more liberal threshold (hemoglobin <9 g/dl) in patients with septic shock (122). This multicenter trial found that patients in the conservative group received a median of 1 unit of pRBCs compared to 4 units in the liberal group, and that there was no difference in mortality or ischemic events in either group (3). This suggests that a lower transfusion threshold is reasonable in patients with septic shock.

Fresh frozen plasma (FFP) is not indicated in patients with sepsis unless a concurrent coagulopathy with evidence of bleeding is present. Interestingly, FFP transfusion has been found to decrease both TNF-α and sydencan-1 levels in critically ill human patients, 45% of which had sepsis (123). This suggests that FFP may decrease both inflammation and endothelial glycocalyx degradation. Although FFP may potentially have a role in disease states with altered endothelial glycocalyx integrity, the clinical implications of this are unknown, and further research is warranted.

## Conclusion

Sepsis is a complex syndrome that may result in vascular alterations, hypoalbuminemia, and myocardial dysfunction, complicating fluid therapy decisions. Fluid therapy plans should be individualized to each patient based on their clinical condition, taking into consideration whether or not there is evidence of hypoperfusion, vasoplegia, hypoalbuminemia, or cardiac dysfunction present. Balanced crystalloid solutions are the mainstay of fluid therapy, although care must be taken to limit edema formation. In patients that are not fluid responsive, norepinephrine should be considered early. Synthetic colloids are not recommended due to the risk of renal injury. If profound hypoalbuminemia and hypotension are present, albumin transfusion can be considered.

## Author Contributions

FM contributed the pathophysiology section. BL contributed the definitions, implications for fluid therapy, and recommendations sections. All authors listed have made a substantial, direct and intellectual contribution to the work, and approved it for publication.

## Conflict of Interest

The authors declare that the research was conducted in the absence of any commercial or financial relationships that could be construed as a potential conflict of interest.

## References

[1] Singer M Deutschman CS Seymour CW Shankar-Hari M Annane D Bauer M. The Third International Consensus Definitions for Sepsis and Septic Shock (Sepsis-3). JAMA. (2016) 315:801–10. 10.1001/jama.2016.0287 26903338 PMC4968574

[2] Tiru B DiNino EK Orenstein A Mailloux PT Pesaturo A Gupta A. The economic and humanistic burden of severe sepsis. Pharmacoeconomics. (2015) 33:925–37. 10.1007/s40273-015-0282-y 25935211

[3] Bentley AM Otto CM Shofer FS. Comparison of dogs with septic peritonitis: 1988–1993 versus 1999–2003. J Vet Emerg Crit Care. (2007) 17:391–8. 10.1111/j.1476-4431.2007.00251.x

[4] Hosgood G Salisbury SK. Generalized peritonitis in dogs: 50 cases (1975–1986). J Am Vet Med Assoc. (1988) 193:1448–50. 3209463

[5] Winkler KP Greenfield CL. Potential prognostic indicators in diffuse peritonitis treated with open peritoneal drainage in the canine patient. J Vet Emerg Crit Care. (2000) 10:259–65. 10.1111/j.1476-4431.2000.tb00011.x

[6] Kenney EM Rozanski EA Rush JE de Laforcade-Buress AM Berg JR Silverstein DC. Association between outcome and organ system dysfunction in dogs with sepsis: 114 cases (2003–2007). J Am Vet Med Assoc. (2010) 236:83–7. 10.2460/javma.236.1.83 20043806

[7] Troìa R Mascalzoni G Calipa S Magagnoli I Dondi F Giunti M. Multiorgan dysfunction syndrome in feline sepsis: prevalence and prognostic implication. J Feline Med Surg. (2019) 21:559–65. 10.1177/1098612X18792106 30099963 PMC10814542

[8] Kaukonen KM Bailey M Pilcher D Cooper DJ Bellomo R. Systemic inflammatory response syndrome criteria in defining severe sepsis. N Engl J Med. (2015) 372:1629–38. 10.1056/NEJMoa1415236 25776936

[9] Hauptman JG Walshaw R Olivier NB. Evaluation of the sensitivity and specificity of diagnostic criteria for sepsis in dogs. Vet Surg. (1997) 26:393–7. 10.1111/j.1532-950x.1997.tb01699.x 9381665

[10] Brady CA Otto CM Van Winkle TJ King LG. Severe sepsis in cats: 29 cases (1986–1998). J Am Vet Med Assoc. (2000) 217:531–5. 10.2460/javma.2000.217.531 10953718

[11] Brubaker SW Bonham KS Zanoni I Kagan JC. Innate immune pattern recognition: a cell biological perspective. Annu Rev Immunol. (2015) 33:257–90. 10.1146/annurev-immunol-032414-112240 25581309 PMC5146691

[12] Kawai T Akira S. The role of pattern-recognition receptors in innate immunity: update on Toll-like receptors. Nat Immunol. (2010) 11:373–84. 10.1038/ni.1863 20404851

[13] Medzhitov R Janeway CA Jr. Innate immunity: impact on the adaptive immune response. Curr Opin Immunol. (1997) 9:4–9. 10.1016/s0952-7915(97)80152-5 9039775

[14] Mogensen TH. Pathogen recognition and inflammatory signaling in innate immune defenses. Clin Microbiol Rev. (2009) 22:240–73. 10.1128/CMR.00046-08 19366914 PMC2668232

[15] Walsh D McCarthy J O'Driscoll C Melgar S. Pattern recognition receptors–molecular orchestrators of inflammation in inflammatory bowel disease. Cytokine Growth Factor Rev. (2013) 24:91–104. 10.1016/j.cytogfr.2012.09.003 23102645

[16] Medzhitov R. Innate immunity: quo vadis? Nat Immunol. (2010) 11:551–3. 10.1038/ni0710-551 20562835

[17] Hauser CJ Otterbein LE. Danger signals from mitochondrial DAMPS in trauma and post-injury sepsis. Eur J Trauma Emer Surg. (2018) 44:317–24. 10.1007/s00068-018-0963-2 29797026

[18] Gong T Liu L Jiang W Zhou R. DAMP-sensing receptors in sterile inflammation and inflammatory diseases. Nat Rev Immunol. (2020) 20:95–112. 10.1038/s41577-019-0215-7 31558839

[19] Tan X Sun L Chen J Chen ZJ. Detection of microbial infections through innate immune sensing of nucleic acids. Annu Rev Microbiol. (2018) 72:447–78. 10.1146/annurev-micro-102215-095605 30200854

[20] Place DE Kanneganti TD. Recent advances in inflammasome biology. Curr Opin Immunol. (2018) 50:32–8. 10.1016/j.coi.2017.10.011 29128729 PMC5857399

[21] Gazendam RP van de Geer A Roos D van den Berg TK Kuijpers TW. How neutrophils kill fungi. Immunol Rev. (2016) 273:299–311. 10.1111/imr.12454 27558342

[22] Satoh T Akira S. Toll-like receptor signaling and its inducible proteins. Microbiol Spectr. (2016) 4:1–7. 10.1128/microbiolspec.MCHD-0040-2016 28084212

[23] Corridoni D Simmons A. Innate immune receptors for cross-presentation: the expanding role of NLRs. Mol Immunol. (2019) 113:6–10. 10.1016/j.molimm.2017.11.028 29198621 PMC6859786

[24] Hamzeh-Cognasse H Berthelot P Tardy B Pozzetto B Bourlet T Laradi S. Platelet toll-like receptors are crucial sensors of infectious danger moieties. Platelets. (2018) 29:533–40. 10.1080/09537104.2018.1445842 29533683

[25] Kumar V. The complement system, toll-like receptors and inflammasomes in host defense: three musketeers' one target. Int Rev Immunol. (2019) 38:131–56. 10.1080/08830185.2019.1609962 31066339

[26] Akira S Uematsu S Takeuchi O. Pathogen recognition and innate immunity. Cell. (2016) 124:783–801. 10.1016/j.cell.2006.02.015 16497588

[27] Janeway CA Jr. Medzhitov R. 2002. Innate immune recognition. Annu Rev Immunol. (2002) 20:197–216. 10.1146/annurev.immunol.20.083001.084359 11861602

[28] O'Callaghan AA Corr SC. Establishing boundaries: the relationship that exists between intestinal epithelial cells and gut-dwelling bacteria. Microorganisms. (2019) 7:663. 10.3390/microorganisms7120663 31818022 PMC6956261

[29] Skibsted S Bhasin MK Henning DJ Jaminet SC Lewandowski J Kirkegaard H. Leukocyte transcriptional response in sepsis. Shock. (2019) 52:166–73. 10.1097/SHK.0000000000001258 30211758 PMC10608800

[30] Weighardt H Heidecke CD Westerholt A Emmanuilidis K Maier S Veit M. Sepsis after major visceral surgery is associated with sustained and interferon-gamma-resistant defects of monocyte cytokine production. Surgery. (2000) 127:309–15. 10.1067/msy.2000.104118 10715987

[31] Newton K Dixit VM. Signaling in innate immunity and inflammation. Cold Spring Harb Perspect Biol. (2012) 4:a006049. 10.1101/cshperspect.a006049 22296764 PMC3282411

[32] Nedeva C Menassa J Puthalakath H. Sepsis: inflammation is a necessary evil. Front Cell Dev Biol. (2019) 7:108. 10.3389/fcell.2019.00108 31281814 PMC6596337

[33] Tsukamoto T Chanthaphavong RS Pape HC. Current theories on the pathophysiology of multiple organ failure after trauma. Injury. (2010) 41:21–6. 10.1016/j.injury.2009.07.010 19729158

[34] Evavold CL Kagan JC. Inflammasomes: threat-assessment organelles of the innate immune system. Immunity. (2019) 51:609–24. 10.1016/j.immuni.2019.08.005 31473100 PMC6801093

[35] Iyer SS Pulskens WP Sadler JJ Butter LM Teske GJ Ulland TK. Necrotic cells trigger a sterile inflammatory response through the Nlrp3 inflammasome. Proc Natl Acad Sci USA. (2009) 106:20388–93. 10.1073/pnas.0908698106 19918053 PMC2787135

[36] Krakauer T. Inflammasomes, autophagy, and cell death: the trinity of innate host defense against intracellular bacteria. Mediators Inflamm. (2019) 2019:2471215. 10.1155/2019/2471215 30728749 PMC6341260

[37] Franchi L Munoz-Planillo R Nunez G. Sensing and reacting to microbes through the inflammasomes. Nat Immunol. (2012) 13:325–32. 10.1038/ni.2231 22430785 PMC3449002

[38] Pittman K Kubes P. Damage-associated molecular patterns control neutrophil recruitment. J Innate Immun. (2013) 5:315–23. 10.1159/000347132 23486162 PMC6741494

[39] Kolaczkowska E Kubes P. Neutrophil recruitment and function in health and inflammation. Nat Rev Immunol. (2013) 13:159–75. 10.1038/nri3399 23435331

[40] Denning NL Aziz M Gurien SD Wang P. DAMPs and NETs in sepsis. Front Immunol. (2019) 10:2536. 10.3389/fimmu.2019.02536 31736963 PMC6831555

[41] Chan BD Wong WY Lee MM Cho WC Yee BK Kwan YW. Exosomes in inflammation and inflammatory disease. Proteomics. (2019) 19:1800149. 10.1002/pmic.201800149 30758141

[42] Stearns-Kurosawa DJ Osuchowski MF Valentine C Kurosawa S Remick DG. The pathogenesis of sepsis. Annu Rev Pathol. (2011) 6:19–48. 10.1146/annurev-pathol-011110-130327 20887193 PMC3684427

[43] Carcillo JA Shakoory B. Cytokine storm and sepsis-induced multiple organ dysfunction syndrome. In: Cron R Behrens EM editors. Cytokine Storm Syndrome. Cham: Springer International Publishing (2019). p. 451–64.

[44] Goggs R Letendre JA. Evaluation of the host cytokine response in dogs with sepsis and noninfectious systemic inflammatory response syndrome. J Vet Emerg Crit Care. (2019) 29:593–603. 10.1111/vec.12903 31637812

[45] Lotze MT Tracey KJ. High-mobility group box 1 protein (HMGB1): nuclear weapon in the immune arsenal. Nat Rev Immunol. (2005) 5:331–42. 10.1038/nri1594 15803152

[46] Charoensup J Sermswan RW Paeyao A Promakhejohn S Punasee S Chularari C. High HMGB1 level is associated with poor outcome of septicemic melioidosis. Int J Infect Dis. (2014) 28:111–6. 10.1016/j.ijid.2014.07.025 25263503

[47] Leisewitz A Goddard A De Gier J Van Engelshoven J Clift S Thompson P. Disease severity and blood cytokine concentrations in dogs with natural *Babesia rossi* infection. Parasite Immunol. (2019) 41:e12630. 10.1111/pim.12630 31063593

[48] Hotchkiss RS Monneret G Payen D. Sepsis-induced immunosuppression: from cellular dysfunctions to immunotherapy. Nat Rev Immunol. (2013) 13:862–74. 10.1038/nri3552 24232462 PMC4077177

[49] Berlot G Passero S. Immunoparalysis in septic shock patients. In: Infectious Process and Sepsis. London: IntechOpen (2019). Available online at: https://www.intechopen.com/books/infectious-process-and-sepsis/immunoparalysis-in-septic-shock-patients (accessed March 2, 2020).

[50] Aird WC. The role of the endothelium in severe sepsis and multiple organ dysfunction syndrome. Blood. (2003) 101:3765–77. 10.1182/blood-2002-06-1887 12543869

[51] Genga KR Shimada T Boyd JH Walley KR Russell JA. The understanding and management of organism toxicity in septic shock. J Innate Immun. (2018) 10:502–14. 10.1159/000487818 29763894 PMC6785648

[52] Lambris JD Ricklin D Geisbrecht BV. Complement evasion by human pathogens. Nat Rev Microbiol. (2008) 6:132–42. 10.1038/nrmicro1824 18197169 PMC2814840

[53] Kerr H Richards A. Complement-mediated injury and protection of endothelium: lessons from atypical haemolytic uraemic syndrome. Immunobiology. (2012) 217:195–203. 10.1016/j.imbio.2011.07.028 21855165 PMC4083254

[54] Brooimans RA Van der Ark AA Tomita M Van Es LA Daha MR. CD59 expressed by human endothelial cells functions as a protective molecule against complement-mediated lysis. Eur J Immunol. (1992) 22:791–7. 10.1002/eji.1830220324 1372260

[55] Rittirsch D Redl H Huber-Lang M. Role of complement in multiorgan failure. Clin Dev Immunol. (2012) 2012:962927. 10.1155/2012/962927 23320021 PMC3539671

[56] McPhaden AR Whaley K. The complement system in sepsis and trauma. Br Med Bull. (1985) 41:281–6. 10.1093/oxfordjournals.bmb.a072063 4027552

[57] Chang JC. Sepsis and septic shock: endothelial molecular pathogenesis associated with vascular microthrombotic disease. Thromb J. (2019) 17:10. 10.1186/s12959-019-0198-4 31160889 PMC6542012

[58] Stiel L Meziani F Helms J. Neutrophil activation during septic shock. Shock. (2018) 49:371–84. 10.1097/SHK.0000000000000980 28858142

[59] Lush CW Kvietys PR. Microvascular dysfunction in sepsis. Microcirculation. (2000) 7:83–101. 10.1038/sj.mn.7300096 10802851

[60] Skoutelis AT Kaleridis V Athanassiou GM Kokkinis KI Missirlis YF Bassaris HP. Neutrophil deformability in patients with sepsis, septic shock, and adult respiratory distress syndrome. Crit Care Med. (2000) 28:2355–9. 10.1097/00003246-200007000-00029 10921564

[61] Uhl B Vadlau Y Zuchtriegel G Nekolla K Sharaf K Gaertner F. Aged neutrophils contribute to the first line of defense in the acute inflammatory response. Blood. (2016) 28:2327–37. 10.1182/blood-2016-05-718999 27609642 PMC5122310

[62] Engelmann B Massberg S. Thrombosis as an intravascular effector of innate immunity. Nat Rev Immunol. (2013) 13:34–45. 10.1038/nri3345 23222502

[63] Ma R Xie R Yu C Si Y Wu X Zhao L. Phosphatidylserine-mediated platelet clearance by endothelium decreases platelet aggregates and procoagulant activity in sepsis. Sci Rep. (2017) 7:4978. 10.1038/s41598-017-04773-8 28694452 PMC5504060

[64] Huang M Cai S Su J. The pathogenesis of sepsis and potential therapeutic targets. Int J Mol Sci. (2019) 20:5376. 10.3390/ijms20215376 31671729 PMC6862039

[65] Mantovani A Cassatella MA Costantini C Jaillon S. Neutrophils in the activation and regulation of innate and adaptive immunity. Nat Rev Immunol. (2011) 11:519–31. 10.1038/nri3024 21785456

[66] Greco E Lupia E Bosco O Vizio B Montrucchio G. Platelets and multi-organ failure in sepsis. Int J Mol Sci. (2017) 18:2200. 10.3390/ijms18102200 29053592 PMC5666881

[67] Lipinska-Gediga M. Platelets in sepsis–are there any new aspects? Anaesthesiol Intensive Ther. (2017) 49:167–72. 10.5603/AIT.a2017.0025 28513821

[68] McDonald B Dunbar M. Platelets and intravascular immunity: guardians of the vascular space during bloodstream infections and sepsis. Front Immunol. (2019) 10:2400. 10.3389/fimmu.2019.02400 31681291 PMC6797619

[69] Monneret G Lepape A Voirin N Bohé J Venet F Debard AL. Persisting low monocyte human leukocyte antigen-DR expression predicts mortality in septic shock. Intensive Care Med. (2006) 32:1175–83. 10.1007/s00134-006-0204-8 16741700

[70] Yao YM Luan YY Zhang QH Sheng ZY. Pathophysiological aspects of sepsis: an overview. Methods Mol Biol. (2015) 1237:5–15. 10.1007/978-1-4939-1776-1_2 25319775

[71] Boomer JS To K Chang KC Takasu O Osborne DF Walton AH. Immunosuppression in patients who die of sepsis and multiple organ failure. JAMA. (2011) 306:2594–605. 10.1001/jama.2011.1829 22187279 PMC3361243

[72] van der Poll T van de Veerdonk FL Scicluna BP Netea MG. The immunopathology of sepsis and potential therapeutic targets. Nat Rev Immunol. (2017) 17:407–20. 10.1038/nri.2017.36 28436424

[73] Abrams ST Morton B Alhamdi Y Alsabani M Lane S Welters ID. A novel assay for neutrophil extracellular traps (NETs) formation independently predicts disseminated intravascular coagulation and mortality in critically ill patients. Am J Respir Crit Care Med. (2019) 200:869–80. 10.1164/rccm.201811-2111OC 31162936 PMC6812439

[74] Patera AC Drewry AM Chang K Beiter ER Osborne D Hotchkiss RS. Frontline Science: defects in immune function in patients with sepsis are associated with PD-1 or PD-L1 expression and can be restored by antibodies targeting PD-1 or PD-L1. J Leukoc Biol. (2016) 100:1239–54. 10.1189/jlb.4HI0616-255R 27671246 PMC5110001

[75] Vallabhajosyula S Kumar M Pandompatam G Sakhuja A Kashyap R Kashani K. Prognostic impact of isolated right ventricular dysfunction in sepsis and septic shock: an 8-year historical cohort study. Ann Intensive Care. (2017) 7:94. 10.1186/s13613-017-0319-9 28884343 PMC5589718

[76] Jardin F Fourme T Page B Loubières Y Vieillard-Baron A Beauchet A. Persistent preload defect in severe sepsis despite fluid loading: a longitudinal echocardiographic study in patients with septic shock. Chest. (1999) 116:1354–9. 10.1378/chest.116.5.1 10559099

[77] Boyd JH Mathur S Wang Y Bateman RM Walley KR. Toll-like receptor stimulation in cardiomyoctes decreases contractility and initiates an NF-kappaB dependent inflammatory response. Cardiovasc Res. (2006) 72:384–93. 10.1016/j.cardiores.2006.09.011 17054926

[78] Boyd JH Kan B Roberts H Wang Y Walley KR. S100A8 and S100A9 mediate endotoxin-induced cardiomyocyte dysfunction via the receptor for advanced glycation end products. Circ Res. (2008) 102:1239–46. 10.1161/CIRCRESAHA.107.167544 18403730

[79] Davani EY Boyd JH Dorscheid DR Wang Y Meredith A Chau E. Cardiac ICAM-1 mediates leukocyte-dependent decreased ventricular contractility in endotoxemic mice. Cardiovasc Res. (2006) 72:134–42. 10.1016/j.cardiores.2006.06.029 16934241

[80] Davani EY Dorscheid DR Lee CH van Breemen C Walley KR. Novel regulatory mechanism of cardiomyocyte contractility involving ICAM-1 and the cytoskeleton. Am J Physiol Heart Circ Physiol. (2004) 287:H1013–22. 10.1152/ajpheart.01177.2003 15087287

[81] Hoesel LM Niederbichler AD Schaefer J Ipaktchi KR Gao H Rittirsch D. C5a-blockade improves burn-induced cardiac dysfunction. J Immunol. (2007) 178:7902–10. 10.4049/jimmunol.178.12.7902 17548628

[82] Fattahi F Frydrych LM Bian G Kalbitz M Herron TJ Malan EA. Role of complement C5a and histones in septic cardiomyopathy. Mol Immunol. (2018) 102:32–41. 10.1016/j.molimm.2018.06.006 29914696 PMC6139045

[83] Marik P Bellomo R. A rational approach to fluid therapy in sepsis. Br J Anaesth. (2016) 116:339–49. 10.1093/bja/aev349 26507493

[84] Uchimido R Schmidt EP Shapiro NI. The glycocalyx: a novel diagnostic and therapeutic target in sepsis. Crit Care. (2019) 23:16. 10.1186/s13054-018-2292-6 30654825 PMC6337861

[85] Puskarich MA Cornelius DC Tharp J Nandi U Jones AE. Plasma syndecan-1 levels identify a cohort of patients with severe sepsis at high risk for intubation after large-volume intravenous fluid resuscitation. J Crit Care. (2016) 36:125–9. 10.1016/j.jcrc.2016.06.027 27546760 PMC6371967

[86] Vincent JL De Backer D Wiedermann CJ. Fluid management in sepsis: the potential beneficial effects of albumin. J Crit Care. (2016) 35:161–7. 10.1016/j.jcrc.2016.04.019 27481753

[87] Walley KR. Sepsis-induced myocardial dysfunction. Curr Opin Crit Care. (2018) 24:292–9. 10.1097/MCC.0000000000000507 29846206

[88] Rivers E Nguyen B Havstad S Ressler J Muzzin A Knoblich B. Early goal-directed therapy in the treatment of severe sepsis and septic shock. N Engl J Med. (2001) 345:1368–77. 10.1056/NEJMoa010307 11794169

[89] Dellinger RP Carlet JM Masur H Gerlach H Calandra T Cohen J. Surviving sepsis campaign guidelines for management of severe sepsis and septic shock. Crit Care Med. (2004) 32:858–73. 10.1097/01.ccm.0000117317.18092.e4 15090974

[90] ProCESS Investigators Yealy DM Kellum JA Huang DT Barnato AE Weissfeld LA. A randomized trial of protocol-based care for early septic shock. N Engl J Med. (2014) 370:1683–93. 10.1056/NEJMoa1401602 24635773 PMC4101700

[91] ARISE Investigators, ANZICS Clinical Trials Group Peake SL Delaney A Bailey M Bellomo R. Goal-directed resuscitation for patients with early septic shock. N Engl J Med. (2014) 371:1496–506. 10.1056/NEJMoa1404380 25272316

[92] Mouncey PR Osborn TM Power GS Harrison DA Sadique MZ Grieve RD. Trial of early, goal-directed resuscitation for septic shock. N Engl J Med. (2015) 372:1301–11. 10.1056/NEJMoa1500896 25776532

[93] Conti-Patara A de Araújo Caldeira J de Mattos-Junior E de Carvalho Hda S Reinoldes A Pedron BG. Changes in tissue perfusion parameters in dogs with severe sepsis/septic shock in response to goal-directed hemodynamic optimization at admission to ICU and the relation to outcome. J Vet Emerg Crit Care. (2012) 22:409–18. 10.1111/j.1476-4431.2012.00769.x 22731982

[94] Rhodes A Evans LE Alhazzani W Levy MM Antonelli M Ferrer R. Surviving sepsis campaign: international guidelines for management of sepsis and septic shock: 2016. Intensive Care Med. (2017) 43:304–77. 10.1007/s00134-017-4683-6 28101605

[95] Levy MM Evans LE Rhodes A. The surviving sepsis campaign bundle: 2018 update. Crit Care Med. (2018) 46:997–1000. 10.1097/CCM.0000000000003119 29767636

[96] Marik PE Farkas JD Spiegel R Weingart S. POINT: should the surviving sepsis campaign guidelines be retired? Yes. Chest. (2019) 155:12–4. 10.1016/j.chest.2018.10.008 30616719

[97] Cavallaro F Sandroni C Marano C La Torre G Mannocci A De Waure C. Diagnostic accuracy of passive leg raising for prediction of fluid responsiveness in adults: systematic review and meta-analysis of clinical studies. Intensive Care Med. (2010) 36:1475–83. 10.1007/s00134-010-1929-y 20502865

[98] Hamzaoui O Georger JF Monnet X Ksouri H Maizel J Richard C. Early administration of norepinephrine increases cardiac preload and cardiac output in septic patients with life-threatening hypotension. Crit Care. (2010) 14:R142. 10.1186/cc9207 20670424 PMC2945123

[99] Li Y Li H Zhang D. Timing of norepinephrine initiation in patients with septic shock: a systematic review and meta-analysis. Crit Care. (2020) 24:488. 10.1186/s13054-020-03204-x 32762765 PMC7409707

[100] Jhanji S Stirling S Patel N Hinds CJ Pearse RM. The effect of increasing doses of norepinephrine on tissue oxygenation and microvascular flow in patients with septic shock. Crit Care Med. (2009) 37:1961–6. 10.1097/CCM.0b013e3181a00a1c 19384212

[101] Koch A Yagmur E Hoss A Buendgens L Herbers U Weiskirchen R. Clinical relevance of copeptin plasma levels as a biomarker of disease severity and mortality in critically ill patients. J Clin Lab Anal. (2018) 32:e22614. 10.1002/jcla.22614 29974524 PMC6817249

[102] Neyra JA Li X Canepa-Escaro F Adams-Huet B Toto RD Yee J. Cumulative fluid balance and mortality in septic patients with or without acute kidney injury and chronic kidney disease. Crit Care Med. (2016) 44:1891–900. 10.1097/CCM.0000000000001835 27352125 PMC5505731

[103] Semler MW Self WH Wanderer JP Ehrenfeld JM Wang L Byrne DW. Balanced crystalloids versus saline in critically ill adults. N Engl J Med. (2018) 378:829–39. 10.1056/NEJMoa1711584 29485925 PMC5846085

[104] Brown RM Wang L Coston TD Krishnan NI Casey JD Wanderer JP. Balanced crystalloids versus saline in sepsis. A secondary analysis of the SMART clinical trial. Am J Respir Crit Care Med. (2019) 200:1487–95. 10.1164/rccm.201903-0557OC 31454263 PMC6909845

[105] Chowdhury AH Cox EF Francis ST Lobo DN. A randomized, controlled, double-blind crossover study on the effects of 2-L infusions of 0.9% saline and plasma-lyte® 148 on renal blood flow velocity and renal cortical tissue perfusion in healthy volunteers. Ann Surg. (2012) 256:18–24. 10.1097/SLA.0b013e318256be72 22580944

[106] Kellum JA Song M Almasri E. Hyperchloremic acidosis increases circulating inflammatory molecules in experimental sepsis. Chest. (2006) 130:962–7. 10.1378/chest.130.4.962 17035425

[107] Santacruz CA De Backer D Taccone FS Su F Orbegozo-Cortes D Hosokawa K. Effects of different crystalloid solutions on hemodynamics, peripheral perfusion, and the microcirculation in experimental abdominal sepsis. Anesthesiology. (2016) 125:744–54. 10.1097/ALN.0000000000001273 27655180

[108] Brunkhorst FM Engel C Bloos F Meier-Hellmann A Ragaller M Weiler N. Intensive insulin therapy and pentastarch resuscitation in severe sepsis. N Engl J Med. (2008) 358:125–39. 10.1056/NEJMoa070716 18184958

[109] Guidet B Martinet O Boulain T Philippart F Poussel JF Maizel J. Assessment of hemodynamic efficacy and safety of 6% hydroxyethylstarch 130/0.4 vs. 0.9% NaCl fluid replacement in patients with severe sepsis: the CRYSTMAS study. Crit Care. (2012) 16:R94. 10.1186/cc11358 22624531 PMC3580640

[110] Perner A Haase N Guttormsen AB Tenhunen J Klemenzson G Åneman A. Hydroxyethyl starch 130/0.42 versus Ringer's acetate in severe sepsis. N Engl J Med. (2012) 367:124–34. 10.1056/NEJMoa1204242 22738085

[111] Mutter TC Ruth CA Dart AB. Hydroxyethyl starch (HES) versus other fluid therapies: effects on kidney function. Cochrane Database Syst Rev. (2013) 7:CD007594. 10.1002/14651858.CD007594.pub3 23881659 PMC11561698

[112] Yozova ID Howard J Adamik KN. Retrospective evaluation of the effects of administration of tetrastarch (hydroxyethyl starch 130/0.4) on plasma creatinine concentration in dogs (2010–2013): 201 dogs. J Vet Emerg Crit Care. (2016) 26:568–77. 10.1111/vec.12483 27144501

[113] Sigrist NE Kälin N Dreyfus A. Changes in serum creatinine concentration and acute kidney injury (AKI) grade in dogs treated with hydroxyethyl starch 130/0.4 from 2013 to 2015. J Vet Intern Med. (2017) 31:434–41. 10.1111/jvim.14645 28109131 PMC5354072

[114] Hayes G Benedicenti L Mathews K. Retrospective cohort study on the incidence of acute kidney injury and death following hydroxyethyl starch (HES 10% 250/0.5/5:1) administration in dogs (2007–2010). J Vet Emerg Crit Care. (2016) 26:35–40. 10.1111/vec.12412 26587795

[115] Sigrist NE Kälin N Dreyfus A. Effects of hydroxyethyl starch 130/0.4 on serum creatinine concentration and development of acute kidney injury in nonazotemic cats. J Vet Intern Med. (2017) 31:1749–56. 10.1111/jvim.14813 28862347 PMC5697200

[116] Yozova ID Howard J Adamik KN. Effect of tetrastarch (hydroxyethyl starch 130/0.4) on plasma creatinine concentration in cats: a retrospective analysis (2010–2015). J Feline Med Surg. (2017) 19:1073–9. 10.1177/1098612X16676160 27803312 PMC11110996

[117] Horowitz FB Read RL Powell LL. A retrospective analysis of 25% human serum albumin supplementation in hypoalbuminemic dogs with septic peritonitis. Can Vet J. (2015) 56:591–7. 26028681 PMC4431157

[118] Artero A Zaragoza R Camarena JJ Sancho S González R Nogueira JM. Prognostic factors of mortality in patients with community-acquired bloodstream infection with severe sepsis and septic shock. J Crit Care. (2010) 25:276–81. 10.1016/j.jcrc.2009.12.004 20149587

[119] Finfer S Bellomo R Boyce N French J Myburgh J Norton R. A comparison of albumin and saline for fluid resuscitation in the intensive care unit. N Engl J Med. (2004) 350:2247–56. 10.1056/NEJMoa040232 15163774

[120] Caironi P Tognoni G Masson S Fumagalli R Pesenti A Romero M. Albumin replacement in patients with severe sepsis or septic shock. N Engl J Med. (2014) 370:1412–21. 10.1056/NEJMoa1305727 24635772

[121] Craft EM Powell LL. The use of canine-specific albumin in dogs with septic peritonitis. J Vet Emerg Crit Care. (2012) 22:631–9. 10.1111/j.1476-4431.2012.00819.x 23216837

[122] Holst LB Haase N Wetterslev J Wernerman J Guttormsen AB Karlsson S. Lower versus higher hemoglobin threshold for transfusion in septic shock. N Engl J Med. (2014) 371:1381–91. 10.1056/NEJMoa1406617 25270275

[123] Straat M Müller MC Meijers JC Arbous MS Spoelstra-de Man AM Beurskens CJ. Effect of transfusion of fresh frozen plasma on parameters of endothelial condition and inflammatory status in non-bleeding critically ill patients: a prospective substudy of a randomized trial. Crit Care. (2015) 19:163. 10.1186/s13054-015-0828-6 25880761 PMC4407778

